# Ascorbic Acid Content and Antioxidant Activities of White and Brown Teff [*Eragrostic tef* (Zucc.)Trotter] Grains and Injera

**DOI:** 10.1155/2023/4751207

**Published:** 2023-03-27

**Authors:** Hagos Yisak, Andargie Belete, Bhagwan Singh Chandravanshi, Mesfin Redi-Abshiro, Estifanos Ele Yaya

**Affiliations:** Department of Chemistry, College of Natural and Computational Sciences, Addis Ababa University, P.O. Box 1176, Addis Ababa, Ethiopia

## Abstract

Teff [*Eragrostis tef* (Zuccagni) Trotter] is a cereal grain originating in Ethiopia as a staple food for millions of people. Its grain is a gluten-free superfood and got acceptance as a medicinal ingredient. Therefore, it is worthwhile to determine the antioxidative activities and L-ascorbic acid contents of teff grain and its baked food (injera). This study aimed to determine the ascorbic acid contents and antioxidant activities in the aqueous extract of the white and brown teff grains and their injera samples using iodimetric titration and UV-Vis spectrophotometric methods, respectively. The ascorbic acid contents in the white and brown teff ranged from 67.9–112.6 mg/100 g and 69.2–117.2 mg/100 g, respectively, and those in injera of the selected teff samples ranged from 30.5–32.9 mg/100 g and 37.3–43.0 mg/100 g, respectively. The antioxidant activities ranged from 1.26–7.04 *μ*mol AAE/g for the white teff grains, 1.44–6.29 *μ*mol AAE/g for the brown teff grains, 1.81–2.47 *μ*mol AAE/g for white teff injera, and 3.89–4.86 *μ*mol AAE/g for the brown teff injera samples. Findings of the present study have revealed that white teff and brown teff grains and their injera were found to have a higher content of ascorbic acid than commonly consumed grains and vegetables. No significant difference (*α* = 0.05) has been observed between the two varieties of teff grains with respect to the ascorbic acid content and antioxidant activities. However, there was a statistically significant difference (*α* = 0.05) in the ascorbic acid content and antioxidant activities between the teff grains and their injera samples. Therefore, this study indicated that teff grains and injera are rich in ascorbic acid content and antioxidant activities as compared to other cereal grains and are very crucial for human nutrition and health.

## 1. Introduction

The need for food security and Ethiopia's diverse climatological and ecological conditions has driven most subsistence farmers to grow various crops [1]. Cereal crops are the staples and the most important source of nutrients for mankind [2]. Teff [*Eragrostis tef* (Zuccagni) Trotter] is a cereal grain originating in Ethiopia as a staple food to millions of people [3]. Only five cereals (rice, wheat, maize, sorghum, and millet) account for more than half of global bread consumption. Excessive use of these selected cereals has the potential to result in genetic losses and difficulty meeting future agricultural demands [4]. Teff offers significantly higher nutritional values than most other cereal grains [5]. It is commercially classified as white and brown cultivars [6]. Teff cultivars outperform all cereal grains in terms of nutritional values, as they contain high levels of carbohydrate, protein, fat, dietary fibers, starch, essential minerals, essential amino acids, vitamins [7–9], polyphenols, volatiles such as aldehydes, ketones, and alcohols, and fatty acids [3]. Teff's global demand is currently increasing due to its healthier nutritional quality, and when compared to other grains, it plays an important role in food security [5, 10]. Furthermore, teff grain is a gluten-free superfood that may benefit people with celiac disease and is gaining acceptance as a medicinal ingredient [3, 5, 9]. Its antioxidative activities, for example, can help prevent malaria and hemoglobin levels in the human body; it can also help prevent diabetes. Furthermore, teff bread has a longer shelf life and a slower aging rate than rice, wheat, maize, sorghum, and barley [9, 11]. Teff can also be used as a fat substitute in producing low-calorie foods due to its high viscosity and low gelling ability [4]. Due to this reason, teff is increasingly studied under the lens of local and international research to support its cultivation and commercialization [12].

Vitamins are essential micronutrients that can be fat-soluble (A, D, E, and K) or water-soluble (B and C) [13, 14]. They are beneficial for the prevention and treatment of various diseases, including heart disease, high cholesterol levels, eye disorders, and skin disorders [7, 11]. Furthermore, vitamins are essential for growth, metabolism, reproduction, and overall health. Dietary vitamin intake is critical except for vitamins D and B1, which the human body cannot synthesize [13].

Niacin, vitamin B6, thiamin, riboflavin, vitamin K (phylloquinone), vitamin A, *α*-tocopherol [7, 15], and vitamin C [4, 7] are all abundant in teff grains. Vitamin C (L-ascorbic acid) has antioxidant properties and anti-inflammatory and antiapoptotic properties. It can also improve the immune system in humans by lowering the body's susceptibility to viral infections. It is one of the most commonly used health supplements to boost immunity and alleviate symptoms caused by COVID-19 infections [16, 17].

Most of plants and animals produce ascorbic acid from D-glucose or D-galactose [18]. L-ascorbic acid, as an antioxidant, lowers the risk of arteriosclerosis, cardiovascular disease, infectious diseases, asthma, cataract, diabetes mellitus, and some types of cancer [19]. It helps relieve common cold symptoms and plays an important role in wound healing. It also prevents free radical oxidation, preventing cell damage, and is commonly used as a food additive [20, 21]. L-ascorbic acid is also necessary to prevent scurvy and maintain healthy skin, gums, and blood vessels. It aids in the formation of collagen, the absorption of inorganic iron, the reduction of plasma cholesterol levels, the inhibition of nitrosoamine formation [21, 22], and the metabolism of tyrosine, folic acid, and tryptophan [21].

L-ascorbic acid less than 300 mg in the body results in scurvy and other disease symptoms. The maximum ascorbic acid in the body is limited to about 2 g for normal health. With high doses of L-ascorbic acid (over 2 g), unabsorbed ascorbate is degraded in the intestine, causing diarrhea. Furthermore, excessive L-ascorbic acid consumption resulted in renal problems, nausea, and gastric irritation [23]. Despite the importance of teff grains for human food and nutritional security, there needs to be more qualitative and quantitative information on vitamins in general, and ascorbic acid in particular in the literature.

The inclusion of antioxidant foods in daily diet is critical to deactivating free radicals [24]. Teff grains have shown better antioxidant potential than other cereals [9]. This makes injera (ready-to-eat food) to be healthy food. Among the two teff varieties, brown teff injera had shown superior antioxidant potentials compared to white teff injera [25].

Various analytical methods for determining ascorbic acid content in fruits and vegetables have been reported in the literature, including potentiometric and reductometric methods [18], high-performance liquid chromatography coupled with ultraviolet spectrophotometry (HPLC-UV) [20, 26], and volumetric and spectrophotometric methods [27, 28]. However, most of these methods are time-consuming, involve multiple chromatographic steps, and require highly skilled technicians, making them problematic. To the best of the researcher's knowledge, no analytical methods for determining L-ascorbic acid contents in cereal grains, specifically teff grain and injera samples, have been reported. As a result, this study aimed to (1) determine the L-ascorbic acid contents in the aqueous extract of the white and brown teff grains and their injera using redox titration with a standardized solution of iodine, (2) evaluate the antioxidant activities of the white and brown teff grains and their injera using the DPPH assay, and (3) correlate the L-ascorbic acid contents and antioxidant activities of the white and brown teff grains.

## 2. Materials and Methods

### 2.1. Apparatus and Instrument

The experiment was carried out using an electronic balance (model: PW254, China) with a precision of 0.0001 g, a grinder (high-speed multifunctional grinder, Shanghai, China), a centrifuge (model: 80-2, China), and a burette set up (10 mL). The absorbance of the prepared standards and sample extracts was measured using a double beam UV-VIS-NIR spectrometer (Lambda 950, Perkin Elmer, UK) with a 1 cm path length quartz cuvette.

### 2.2. Chemicals and Reagents

This study used chemicals such as KIO_3_, KI, L-ascorbic acid (Riede-de Haen, Germany), starch indicators, and 96% H_2_SO_4_ (Carlo Erba, Italy). Throughout the experiment, distilled and deionized water was used.

### 2.3. Sample Collection and Pretreatment

The sample collection and pretreatment were described elsewhere [3, 9].

Injera was made to compare the ascorbic acid content of white and brown teff grains. The traditional fermentation of teff flour was used to prepare injera. Teff flour was combined with the previous batch's water and ersho (without yeast additives). For primary fermentation, the mixture was fermented for 42 hours. A portion of the batter was mixed and boiled after primary fermentation to produce absit (gelatinization process). The prepared absit mixes were added to the primary fermented batter and allowed to ferment for 4 hours.

Finally, the batter was made to injera using Mitad. The prepared injera was dried for four days at room temperature before being ground to mesh size with an electronic grinder, and it was prepared for extraction.

### 2.4. Extraction of Ascorbic Acid in the White and Brown Teff Grains and Injera Samples

Yisak et al. [9] described the adopted method to extract the ascorbic acid content of white and brown teff grains and injera samples. In brief, 0.25 g of ground white teff, brown teff, and injera samples was soaked for 10 min in 20 mL of the extraction solvent (deionized water). Handshaking extracted the ascorbic acid from the wetted samples for 20 minutes, and the mixture was centrifuged for 15 minute at 3000 rpm. Finally, the supernatant was filtered through Whatman filter paper and prepared for analysis.

### 2.5. Preparation of Reagents and Standards

The preparation of 3 M H_2_SO_4_, 0.5% starch indicators and preparation of iodine solution for the titration method were described elsewhere [29].

#### 2.5.1. Preparation of the Ascorbic Acid Standard (100 mg/L) Solution for Standardization in the Titration Method

A 100 mg/L-ascorbic acid standard solution was made by dissolving 0.005 g ascorbic acid (L-ascorbic acid) in a 50 mL volumetric flask with distilled water and then filling the flask to the mark with the solvent.

#### 2.5.2. Standardizing Iodine Solutions for the Titration Method

A 100 mL Erlenmeyer flask was filled with 20 mL of 100 mg/L-ascorbic acid standard solution, and 1 mL of 0.5% starch solution was added. This solution was titrated with a small volume of iodine solution (1.95 mL) until the endpoint was reached. The endpoint was discovered when the first sign of the purple-blue color appeared during the titration process. The initial and final volumes of the iodine solution were then measured. The average concentration of the iodine solution was calculated by repeating titration three times and averaging the three results.

### 2.6. Determination of Ascorbic Acid Content by Iodimetric Titration

Many researchers reported titration as a preferable method for ascorbic acid determination due to its simplicity, low cost, and speed. The ascorbic acid determination method was adopted from Belete et al. [29] and Satpathy et al. [30] with some modifications. The oxidation-reduction reaction was carried out based on iodimetric titration of the sample. To determine the amount of ascorbic acid, 20 mL of the sample extract was taken and 1 mL of 0.5% starch solution was added to each extract.

The solution was titrated against the prepared iodine solution while shaking continuously, and the endpoint for each sample was recorded. The titration was performed in triplicate, and the results were presented as a mean ± SD on a dry basis from triplicate measurements.

#### 2.6.1. Determination of Antioxidant Activities of the White and Brown Teff Grains and Their Injera Samples

The antioxidant activity of the white and brown teff grains and their injera extracts was determined using the 2,2-diphenyl-1-picrylhydrazyl (DPPH) free radical scavenging activity method described by Yisak et al. [9] with some modifications. In a 200 mL volumetric flask, 0.02 g of DPPH was dissolved with a small amount of methanol. After DPPH was completely dissolved, the flask was filled to the mark with methanol to achieve a solution concentration of 253.60 *μ*mol/L. 3 mL of methanol and 2 mL of DPPH solution were used as controls. Furthermore, an ascorbic acid stock solution (1135.6 *μ*mol/L) was prepared by dissolving 0.02 g of ascorbic acid in 100 mL of the volumetric flask with methanol. A calibration curve was established by preparing different concentrations of the stock solution (136.3, 102.2, 68.14, 17.74, and 6.036 *μ*mol/L). A volume of 1 mL of each standard ascorbic acid solution was transferred into five different 25 mL volumetric flasks, and each flask received 3 mL of methanol and 2 mL of DPPH solution before being incubated in the dark at room temperature for 60 min. Finally, the absorbance at 517 nm was measured.

A 1 mL portion of the extract was mixed with 3 mL of methanol and 2 mL of DPPH solution for the samples. For 60 min, the mixture was kept in the dark at room temperature. Using a calibration curve, the results were expressed as micromoles of ascorbic acid equivalent/g on a dry basis (*μ*mol AAE/g). Each sample was scanned three times.

### 2.7. Statistical Analysis

All analyses were performed in triplicate. On a dry matter basis, the results were reported as a mean ± SD. The differences in mean values between teff grain varieties and injera were determined using one-way ANOVA, followed by Tukey's honestly significant difference (HSD) multiple rank test (*α* = 0.05) [31]. Minitab 17 software was used for all statistical analyses.

## 3. Results and Discussion

### 3.1. Determination of Ascorbic Acid Content in Teff

Tables 1 and 2 show the determined ascorbic acid contents of white teff grains and brown teff grains and their injera samples in milligrams per 100 g of flour on a dry basis (mg/100 g dry basis). The ascorbic acid contents of the white and brown teff aqueous extracts were 67.9–112.6 mg/100 g and 69.2–117.2 mg/100 g, respectively, and those in injera of the selected teff samples ranged from 30.5–32.9 mg/100 g and 37.3–43.0 mg/100 g, respectively. As shown in Table 1, the brown teff grain sample from Were Ilu district (South Wollo zone) contained the highest quantity of ascorbic acid. In contrast, the white teff grain sample from Minjar Shenkora district (North Shewa zone) contained the least quantity of all the teff grains analyzed. The ascorbic acid content of teff grains is higher than that of the selected teff injera samples. Because ascorbic acid is susceptible to food-processing procedures and radiation due to its high solubility in water, cooking can affect its content. However, significant losses do not occur with the typical household cooking method [21]. Furthermore, Otemuyiwa et al. [32] described that a combination of leaching and chemical destruction causes vitamin loss during cooking.

Gebremariam et al. [7] reported an ascorbic acid content in teff of 88 mg/100 g, which is within the range of the teff grains and higher than the content in the current study's injera samples. However, the authors did not provide any information about the methods used to determine ascorbic acid in the teff grain samples. In comparison to this study, the ascorbic acid content of raw rice (0.3–1.1 mg/100 g), cooked rice (0.04–0.56 mg/100 g) [32], and barley grains (0.35–0.38 mg/100 g is very low [33]. Furthermore, Satpathy et al. [30] reported ascorbic acid concentrations in some vegetables, including garlic (40.95 mg/100 g), onion (30.79 mg/100 g), potato (33.65 mg/100 g), tomato (16.47 mg/100 g), pea (50.84 mg/100 g), common bean (41.28 mg/100 g), pumpkin (36.30 mg/100 g), and cucumber (24.23 mg/100 g), which are in agreement with the content of ascorbic acid in the selected teff injera samples and lower than the teff grain samples of the present study. Therefore, it is evident from the present findings that one can get enough ascorbic acid in a given teff injera food.

One-way ANOVA (*α* = 0.05) was used to test for the presence of significant differences in the mean concentration of ascorbic acid in white and brown teff grains (Table 1) and their injera samples (Table 2). The ANOVA test revealed no statistically significant differences (*α* = 0.05) between the two teff grain varieties. Still, there was a significant difference (*α* = 0.05) in the mean concentration of ascorbic acid between the teff grains and their injera samples.

### 3.2. Precision and Recovery of the Method

The precision (% RSD) and accuracy (% recovery) of the titration method for determining ascorbic acid content were assessed. As a result, the method's repeatability was evaluated by calculating the relative standard deviation (RSD) of triplicate measurements, which yielded 1.8–7.6%, indicating that the method is precise. The reproducibility (recovery) test was carried out by adding a known amount of ascorbic acid to injera, white, and brown teff extracts. The spiked solution was analyzed three times to obtain the average recovery (*R*), which was calculated using the formula: [(*C*_*S*_ − *C*)/*C*_*A*_] × 100 = %*R*, where *C*_*S*_ represents the concentration of the spiked sample extract, *C* represents the concentration of the unspiked sample extract, and *C*_*A*_ represents the concentration of the spiked ascorbic acid. The percent recovery results (98.6–104%) in Table 3 demonstrate that the method is reproducible for determining ascorbic acid in teff grain and injera extracts.

### 3.3. Determination of Antioxidant Activities of the White and Brown Teff Grains and Their Injera Samples

**Figure 1:**
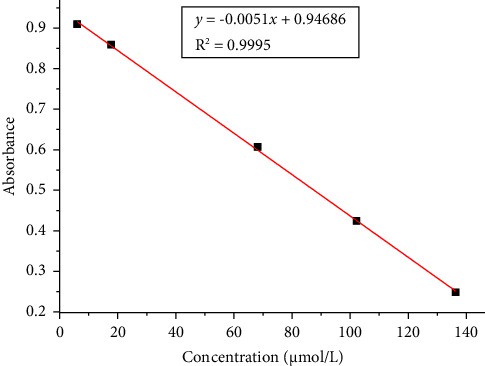
The calibration curve of ascorbic acid standard DPPH scavenging activities.

**Figure 2:**
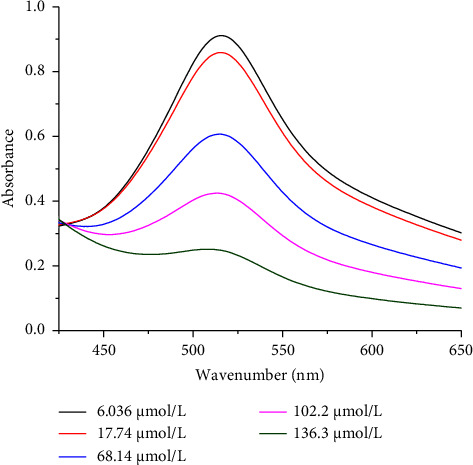
Overlay spectra of ascorbic acid standard DPPH scavenging activities.

The antioxidant capacity of cereal grains can be determined using a variety of assays. The DPPH assay was used in this study to assess the antioxidant activities of the ascorbic acid in white and brown teff grains and their respective injera samples (Table 4).  Figure 1 shows the calibration curve (*y* = −0.0051*x* + 0.94686) of ascorbic acid standard DPPH scavenging activities determined by its regression coefficient (*R*^2^ = 0.9995).  Figure 2 depicts the UV–Vis overlay spectra of ascorbic acid standard DPPH scavenging activities.

The antioxidant activity levels in white and brown teff grains were 1.26–7.04 *μ*mol AAE/g and 1.44–6.29 *μ*mol AAE/g, respectively. The lowest and highest values of DPPH radical scavenging activities in white teff grain samples were determined for Ada'a district (East Shewa zone) and Goncha Siso Enese district (East Gojjam zone), respectively, while those of brown teff grain samples were determined for Dessie Zuria district (South Wollo zone) and Gomibora district (Hadiya zone). Furthermore, the DPPH scavenging activities of white and brown teff injera were found to be 1.81–2.47 *μ*mol AAE/g and 3.89–4.86 *μ*mol AAE/g, respectively.

**Figure 3:**
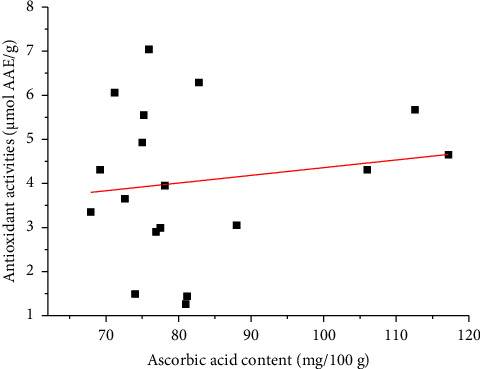
Relationship between ascorbic acid contents and *in vitro* antioxidant activities of the white and brown teff grains.

This study found that the antioxidant activities of white and brown teff grains are comparable to those of the literature reports of other cereal grains presented as *μ*mol of trolox equivalent antioxidant capacities per g of the sample (*μ*mol TE/g sample) like rice (1.39–10 *μ*mol TE/g) [34], wheat (7–10 *μ*mol TE/g) [35], white teff (4.32–6.36 *μ*mol TE/g), and brown teff (6.54–7.16 *μ*mol TE/g) [36], but they are higher than the antioxidant activities of the white teff (2.10–2.14 *μ*mol TE/g) and brown teff grains (2.14–4.30 µmol TE/g) reported by Kotaskova et al. [37]. One-way ANOVA indicated that there was no significant differences (*α* = 0.05) in the mean antioxidant activity content between the white and brown teff grains. The low correlation (*r* = 0.14937) between ascorbic acid contents and in vitro antioxidant activities (Figure 3) in the teff grains may suggest that the major antioxidant compounds in the sample might be bioactive compounds other than ascorbic acid.

The antioxidant activities of some selected white and brown teff injera prepared in 42 hours of fermentation were evaluated using the DPPH assay, as shown in Table 5. As a result, the antioxidant activity content of the injera samples was lower than that of the teff grains using the DPPH method. This is because bioactive compounds such as ascorbic acid, which acts as an antioxidant, are susceptible to cooking and radiation, resulting in a loss of DPPH scavenging activity in the injera samples. The one-way ANOVA test revealed significant differences (*α* = 0.05) between the white and brown teff grains and their injera samples (Table 5).

## 4. Conclusion

The teff's ascorbic acid content is important for human health and is used as a quality indicator parameter. As a result, the study reported ascorbic acid contents and antioxidant activities in aqueous extracts of white teff grains and brown teff grains and their injera samples. The mean ascorbic acid content and antioxidant activities determined in the white and brown teff grains have shown no significant differences (*α* = 0.05). However, the mean concentration of ascorbic acid and antioxidant activities of the selected teff grains and their injera samples differed significantly (*α* = 0.05). According to this study, teff grains and their corresponding injera have high ascorbic acid content and antioxidant activity. Furthermore, the ascorbic acid content of teff grain and injera was higher than that of commonly consumed grains and vegetables. Were Ilu district of the South Wollo zone had the highest ascorbic acid content in white and brown teff grains. To the best of our knowledge, this is the first study to determine the ascorbic acid content of teff grains and injera. As a result, this finding can serve as a foundation for future research on using teff grains in functional foods.

## 5. Recommendations

It is fact that teff grain is typically used in fermented foods. The endogenous and microbial enzymes derived from teff grain flour initiate injera dough fermentation which could be an interesting area of future research. Besides, establishing standards for the quality and quantity of starter culture, fermentation conditions, and other ingredients can let researchers investigate biological activities and prospective applications.

## Figures and Tables

**Table 1:** Ascorbic acid content (mean ± SD) of the white and brown teff [*Eragrostis tef* (Zuccagni) Trotter] grains determined by iodimetric titration.

Region	Administrative zone	District	Variety of teff	Sample ID	Concentration (mg/100 g)
Amhara	North Shewa	Minjar Shenkora	White teff	AW-1	67.9 ± 3.2
			Brown teff	AB-19	88.0 ± 2.3
	South Wollo	Dessie Zuria	White teff	AW-5	72.6 ± 2.8
			Brown teff	AB-23	81.2 ± 5.5
		Were Ilu	White teff	AW-6	112.6 ± 2.0
			Brown teff	AB-24	117.2 ± 5.9
	East Gojjam	Goncha Siso Enese	White teff	AW-8	75.9 ± 1.6
			Brown teff	AB-26	75.0 ± 3.2

Oromia	Arsi	Jeju	White teff	OW-11	74.0 ± 5.6
			Brown teff	OB-29	77.5 ± 1.9
	East Shewa	Ada'a	White teff	OW-13	81.0 ± 3.0
			Brown teff	OB-31	106.0 ± 4.5
		Bishoftu	White teff	OW-14	71.2 ± 2.9
			Brown teff	OB-32	69.2 ± 2.30

SNNPR	Hadiya	Soro	White teff	SW-17	76.9 ± 2.9
			Brown teff	SB-35	78.1 ± 1.6
		Gomibora	White teff	SW-18	75.2 ± 3.6
			Brown teff	SB-36	82.8 ± 2.4

**Table 2:** Comparison of the ascorbic acid content (mean ± SD) in the selected white and brown teff [*Eragrostis tef* (Zuccagni) Trotter)] grains and injera samples.

Sample ID	Variety of teff	Concentration (mg/100 g)	
		Grain	Injera
AW-5	White teff	72.6 ± 2.8^b^	30.5 ± 1.8^d^
AB-23	Brown teff	81.2 ± 5.5^b^	37.3 ± 2.9^cd^
OW-13	White teff	81.0 ± 3.0^b^	32.9 ± 1.5^d^
OB-31	Brown teff	106.0 ± 4.5^a^	43.0 ± 2.9^c^

*Note*. Values (means ± SD, *n* = 3) within a row and column with different superscript letters are significantly different (*α* = 0.05).

**Table 3:** Recovery results of ascorbic acid by the redox titration method.

Variety of sample	Amount of ascorbic acid in the sample before spiking (mg)	Amount of ascorbic acid spiked (mg)	Amount of ascorbic acid found after spiking (mg)	Recovery (%) (*n* = 3)
White teff grain	0.238	0.0952	0.332	98.7 ± 4.3
Brown teff grain	0.218	0.0872	0.304	98.6 ± 5.3
White teff injera	0.088	0.0348	0.124	104 ± 4.9
Brown teff injera	0.083	0.0332	0.117	102 ± 4.3

**Table 4:** DPPH radical scavenging activities in the white and brown teff grains varieties.

Sample region	Variety of teff	Sample ID	Antioxidant activities (*μ*mol AAE/g)
Amhara	White teff	AW-1	3.35 ± 0.36
		AW-5	3.05 ± 0.14
		AW-6	3.65 ± 0.39
		AW-8	1.44 ± 0.21
	Brown teff	AB-19	5.67 ± 0.21
		AB-23	4.65 ± 0.11
		AB-24	7.04 ± 0.36
		AB-26	4.93 ± 0.14

Oromia	White teff	OW-11	1.49 ± 0.25
		OW-13	2.99 ± 0.21
		OW-14	1.26 ± 0.18
	Brown teff	OB-29	4.31 ± 0.15
		OB-31	6.06 ± 0.20
		OB-32	4.31 ± 0.14

SNNPR	White teff	SW-17	2.90 ± 0.25
		SW-18	3.95 ± 0.14
	Brown teff	SB-35	5.55 ± 0.13
		SB-36	6.29 ± 0.28

ID = identification; AAE = ascorbic acid equivalent.

**Table 5:** Comparison of the antioxidant activities in the aqueous extract of white and brown teff grains and their injera samples.

Sample ID	Antioxidant activity (*μ*mol AAE/g)	
	Grain	Injera
AW-5	3.05 ± 0.14^d^	2.47 ± 0.28^de^
AB-23	4.65 ± 0.11^b^	3.89 ± 0.13^c^
OW-13	2.99 ± 0.21^d^	1.81 ± 0.33^e^
OB-31	6.06 ± 0.20^a^	4.86 ± 0.33^b^

ID = identification; AAE = ascorbic acid equivalent. Values (means ± SD, *n* = 3) within a row and column with different superscript letters are significantly different (*α* = 0.05).

## Data Availability

All the data are included in the manuscript. There are no additional data with the authors.
